# Supplementary material for: Protective Effect of Foxtail Millet Protein Hydrolysate on Ethanol and Pyloric Ligation-Induced Gastric Ulcers in Mice
Source: Antioxidants (Basel). 2022 Dec 14;11(12):2459. doi: 10.3390/antiox11122459 (PMC9774519; doi:10.3390/antiox11122459)
Supplement: Supplementary file 1 [file antioxidants-11-02459-s001.zip › antioxidants-2076131-Supplementary-Table S1.pdf]

Supplementary Data

Protective effect of foxtail millet protein hydrolyzate on ethanol and pyloric ligation-induced gastric ulcer in mice

Table: 2

Table S1 Amino acid composition of FMPH

Amino acid (%)	
Leu[a]	11.3 ± 0.03
Ala[a]	7.29 ± 0.05
Pro[a]	6.47 ± 0.21
Phe[a]	4.73 ± 0.08
Val[a]	4.09 ± 0.06
Ile[a]	3.47 ± 0.07
Tyr[a]	2.49 ± 0.21
Met[a]	2.16 ± 0.09
Glu	15.9 ± 0.09
Asp	6.61 ± 0.03
Ser	4.01 ± 0.13
Thr	3.22 ± 0.12
Arg	2.79 ± 0.14
Gly	2.74 ± 0.12
His	2.10 ± 0.06
Trp	1.69 ± 0.09
Cys	1.03 ± 0.11
Lys	1.51 ± 0.07
Total	83.6 ± 1.76

[a]Hydrophobic amino acids (HAA): Leu, Ala, Pro, Phe, Val, Ile, Tyr, and Met.
